# Is invasion success of Australian trees mediated by their native biogeography, phylogenetic history, or both?

**DOI:** 10.1093/aobpla/plw080

**Published:** 2016-12-30

**Authors:** Joseph T. Miller, Cang Hui, Andrew H. Thornhill, Laure Gallien, Johannes J. Le Roux, David M. Richardson

**Affiliations:** 1 National Research Collections Australia, CSIRO National Facilities and Collections, GPO Box 1600, Canberra, ACT 2601, Australia; 2 Office of International Science and Engineering, National Science Foundation, Arlington, Virginia 22230, USA; 3 Department of Mathematical Sciences, Centre for Invasion Biology, Stellenbosch University, Matieland, 7620 South Africa; 4 BioMath Group, African Institute for Mathematical A10 Sciences, Cape Town, 7652 South Africa; 5 Department of Integrative Biology, University and Jepson Herbaria and University of California, Berkeley, CA 94720-2465, USA; 6 Department of Botany and Zoology, Centre for Invasion Biology, Stellenbosch University, Matieland, 7620 South Africa

**Keywords:** Acacia, eucalypts invasiveness, introduced, naturalized, phylogenetic signal, spatial clustering, tree invasions

## Abstract

For a plant species to become invasive it has to progress along the introduction-naturalization-invasion (INI) continuum which reflects the joint direction of niche breadth. Identification of traits that correlate with and drive species invasiveness along the continuum is a major focus of invasion biology. If invasiveness is underlain by heritable traits, and if such traits are phylogenetically conserved, then we would expect non-native species with different introduction status (i.e. position along the INI continuum) to show phylogenetic signal. This study uses two clades that contain a large number of invasive tree species from the genera *Acacia* and *Eucalyptus* to test whether geographic distribution and a novel phylogenetic conservation method can predict which species have been introduced, became naturalized, and invasive. Our results suggest that no underlying phylogenetic signal underlies the introduction status for both groups of trees, except for introduced acacias. The more invasive acacia clade contains invasive species that have smoother geographic distributions and are more marginal in the phylogenetic network. The less invasive *Eucalyptus* group contains invasive species that are more clustered geographically, more centrally located in the phylogenetic network and have phylogenetic distances between invasive and non-invasive species that are trending toward the mean pairwise distance. This suggests that highly invasive groups may be identified because they have invasive species with smoother and faster expanding native distributions and are located closer to the edges of phylogenetic networks than less invasive groups.

## Teaser

Two speciose clades of Australian trees, Acacia and Eucalyptus, have shown differential invasive ability when exported, with introduced Acacia species more likely to become invasive. The evolutionary histories of Acacia and Eucalyptus clades do not contain underlying phylogenetic signals for invasiveness as individual component species progress along the introduction-naturalization-invasion (INI) continuum. Geographic and network analyses suggest that potentially invasive clades, like Acacia, may be identified because they contain invasive species with smoother and faster expanding native distributions and are located more to the edges of phylogenetic networks than less invasive clades.

## Introduction

In order to anticipate which introduced species might become invasive it is crucial to identify those characteristics that are correlated with, and potentially drive, species overcoming the so-called introduction-naturalization-invasion (INI) continuum (Blackburn *et al.* 2011; Richardson and Pyšek 2012; Hui *et al.* 2013). To progress along the INI continuum introduced species need to sequentially pass through a number of barriers (e.g. dispersal, environmental and biotic), and thus the continuum reflects the niche breadth. However, factors driving the evolutionary history and native range structures of introduced species could be analogous, but not identical, to factors/traits of species invasiveness and performance in introduced ranges (Zenni *et al.* 2017). Consequently, examining the phylogenetic signals and native range structures of large clades containing species differing in their advancement along the INI continuum could help to pinpoint the common evolutionary and geographical features of successful invaders.

The Australian flora has evolved under unique circumstances. The continent has been drifting away from other large landmasses for 50 million years, which has limited dispersal to and from Australia (Crisp and Cook 2013). This has led to the evolution of large and unique flora, such as wattles (genus *Acacia*), eucalypts (genera *Angophora*, *Corymbia* and *Eucalyptus*) and several lineages of the Proteaceae such as members of the *Banksia* and the *Grevillea/Hakea* clades, that are wholly, or almost exclusively, native to the continent. These lineages evolved during large-scale environmental fluctuations that particularly fostered their diversifications, notably during relatively recent periods of aridification (over the last 8–10 million years; Byrne *et al.* 2008).

Biogeographic isolation and high resistance to environmental fluctuations have together made some of the highly diverse flora extremely well adapted for survival, growth and proliferation in many other parts of the world (Richardson *et al.* 2011). Indeed, many Australian plants, including hundreds of species of *Acacia* and eucalypts, have been moved to many areas of the world for the past 150 years (most notably to South Africa, the Americas, southern Europe) to perform supporting and provisioning ecosystem services such as soil erosion control, wood, perfume or leather tannins production (Reichard and Hamilton 1997; Wilson *et al.* 2011). Unfortunately, several introduced Australian species have also recently been recognized as some of the world’s most invasive and environmentally devastating invasive species (Lowe *et al.* 2000; Pejchar and Mooney 2009).

The purposeful introduction of species from multiple long-isolated Australian clades into other parts of the world with similar climatic conditions, allows a comparative framework to investigate which aspects, such as life-history traits, evolutionary history and/or environmental niche, are critical to the succession along the INI continuum. To date, tree height and native range size (also the range of mean annual precipitation) have been identified as strong predictors of invasiveness for Australian *Acacia* species (Gallagher *et al.* 2011), while, to our knowledge, no consensus has been reached for Australian eucalypts.

**Figure 1:**
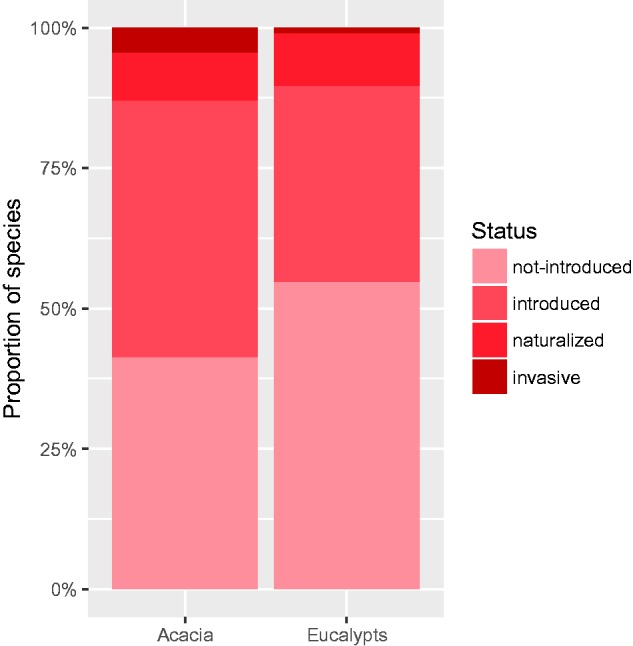
Percentage of species diversity introduced outside Australia that is invasive or naturalized outside Australia. Data are given as percentage of species.

Acacias, and legumes in general, are more invasive than the eucalypts (larger proportion of invasive species and invaded areas; Rejmánek and Richardson 2011, 2013). Although the eucalypt clade contains a slightly larger proportion of naturalized species (9.3 vs. 8.6 %), the *Acacia* clade includes more than four times more invasive species (4.3 vs. 1 %; Fig. 1). Both clades contain hundreds of species (489 *Acacia* spp. and 711 eucalypts in this study), many of which have been introduced outside Australia (287 *Acacia* spp. and 322 eucalypts in this study). However, the question of why *Acacia* species are generally more invasive than eucalypt species and why some eucalypts are more invasive than others remains unanswered. Comparing the evolutionary history of the *Acacia* and eucalypt clades along the INI continuum may shed light on why only some of the species have successfully progressed further along the continuum.

One key opportunity to explore the differences between these two clades is to take advantage of their recently reconstructed multi-gene phylogenies (Mishler *et al.* 2014; González-Orozco *et al.* 2016). Indeed, detailed phylogenies permit investigation of the role of evolutionary history in species invasiveness and to generate clade-specific hypotheses that may also be tested. For instance, Yessoufou *et al.* (2016) recently found that naturalized non-invasive and invasive acacias are not a phylogenetically random subset of taxa when considering all Australian acacias introduced globally. This result suggests that invasive acacias species may have heritable functional traits that can favor their invasiveness (e.g. long dispersal distances, or high resistance to disturbances).

To explain differences in the invasiveness of eucalypts and acacias it has been suggested that human introductions of these species were not random but instead driven by species-specific colonization capacities (Richardson *et al.* 2011). Hui *et al.* (2014) for instance suggested, based on native range size, that introduced *Acacia* species had faster spread rates than eucalypt species. Two reasons were speculated: first, *Acacia* is a younger clade than *Eucalyptus* and could be inherently equipped to rapidly colonize new ranges, whereas the older eucalypts will likely colonize new suitable ranges much more slowly (Hui *et al.* 2014); second, the purpose and history of introduction and propagule pressure (number and size of introduction events) may differ between these two lineages. If invasiveness of eucalypts and *Acacia* species is underlain by heritable traits related to their spread rates, such as dispersal capacity and seed production, and if such traits are phylogenetically conserved, then we would expect the introduction status of a species (e.g. naturalized vs. invasive species) to show a phylogenetic signal. In turn, phylogenetic patterns of invasiveness might be useful to inform risk assessments prior or shortly after introductions to other parts of the world (Gallien *et al.* 2016).

In this study, we utilize detailed native range spatial datasets and near-complete phylogenies of acacias and eucalypts to investigate the role of evolutionary history in the INI continuum. To compare clades of these two groups we estimated their respective phylogenetic signals of introduction status. In particular we ask the following questions. (i) Is there overall phylogenetic signal or spatial aggregation for acacias or eucalypts at any stage of the INI continuum? (ii) Do the attributes, such as standard deviation, median, quantiles, minimum, maximum, skewness and kurtosis, of the mean phylogenetic differences among the three introduction status categories along the INI continuum (introduced, naturalized and invasive) differ between acacias and eucalypts? and (iii) Can these differences in phylogeny and range structure, if any, be interpreted in the context of invasiveness?

## Methods

### *Acacia* and *eucalypts* phylogenies

Separate phylogenies of Australian acacias and eucalypts have been published (Mishler *et al.* 2014; González-Orozco *et al.* 2016). The *Acacia* phylogeny contains 489 *Acacia* species and was inferred from a maximum likelihood analysis of four plastid loci (*psb*A-*trn*H and *rpl*32-*trn*L intergenic spacers, the *trn*L-F intron and intergenic spacer, and a portion of the *mat*K intron) and two nuclear ribosomal DNA loci (ITS, internal transcribed spacers and ETS, external transcribed spacer). The eucalypt phylogeny contains 711 species of *Angophora*, *Corymbia* and *Eucalyptus* inferred from a maximum likelihood analysis of two plastid loci (*matK* and *psbA-trnH*) and the two nuclear ribosomal DNA loci (ITS and ETS).

### Species status along the INI

The status of *Acacia* and *eucalypt* species along the INI continuum (Blackburn *et al.* 2011) was determined based on 30 and nine species lists, respectively (Hui *et al.* 2011, 2014). These sources of information notably included published invasive species lists (Poynton 1979; 2009; Kueffer *et al.* 2010; Castro-Díez *et al.* 2011; Rejmánek and Richardson 2011, 2013), invasive species databases such as the Rod Randall’s Global Compendium of Weeds (GCWs; hear.org/gcw), the Southern African Plant Invaders Atlas (agis.agric.z/wip), the European Garden Flora (Cullen *et al.* 2011) or the European Invasive Alien Species Gateway (Europe-aliens.org), national herbaria (the South African herbaria; H. Glen, unpubl. data); and records of seeds dispatched internationally by the Australian Tree Seed Centre (Griffin *et al.*, 2011). Species recorded in more than 10 countries outside Australia in the Global Biodiversity Information Facility database (data.gbif.org) were additionally considered as introduced.

### Phylogenetic signal of species status

To explore the phylogenetic signals of species’ introduction status along the INI we used two methods, a traditional and a more recently developed one. The traditional method for estimating the phylogenetic signal of invasiveness (a binary trait) is based on the mean phylogenetic distance (MPD) between groups of species pairs index. MPD’s significant clustering (i.e. significant phylogenetic signal) compared with null expectations (i.e. the *P*-value) was determined by 999 randomizations of the species introduction status across the species compared in the test (e.g. randomizing the position of invasive acacia in the acacia phylogeny). For both *Acacia* and *eucalypts* we tested phylogenetic signal along the INI continuum with the following pairwise comparisons: introduced vs. all species, naturalized vs. all species, invasive vs. all species, naturalized vs. introduced species, invasive vs. introduced species, and invasive vs. naturalized + invasive species. In each case the phylogenies were trimmed to include only the species under consideration.

Complementarily, we also estimated species phylogenetic signals of species status (introduced, naturalized and invasive) along the INI based on the pairwise genetic distances (Kimura, 1980) from the *Acacia* and *Eucalyptus* DNA sequencing alignments. Phylogenetic trees are but one particular subset of phylogenetic networks, with the latter a broader and sometimes more powerful tool to visualize evolutionary relationships (Huson *et al.* 2010). The matrix of pairwise genetic distance can be used as the weighted adjacency matrix to build such a phylogenetic network. From these pairwise genetic distances we calculated nine metrics for each species: the mean, SD, median, 2.5 and 97.5 % quantiles, minimum, maximum, skewness and kurtosis of its genetic distance to all other species contained in the overall phylogeny. These metrics describe how a given species is related to all other species in the phylogenetic network: the mean and median component depict how ‘central’ a species is located in the phylogenetic network (a species located in the center should have the smallest mean and median), while the 97.5 % quantiles depict how ‘centralized’ a species is in the phylogenetic network. Skewness would suggest an overall trend of one of the introduction status classes to have a pairwise distance distribution that is consistently higher or lower (depending on the direction of the skewness) than the other classes. Kurtosis, by contrast, depicts the peakedness of a normal distribution (a kurtosis < 3 reflects a ‘flat-topped’ distribution, while a kurtosis > 3 reflects a ‘pointy’ distribution). We then performed an analysis of variance (ANOVA) for these metrics as a function of the introduction status (introduced, naturalized and invasive). Mean, median and minimum genetic distances were log-transformed to make their frequency distributions follow a normal-like distribution, with an increment of 0.01 added to the minimum before the transformation.

### Native range sizes and spatial clustering

In previous studies, we obtained over 220 000 herbarium records for acacias (Hui *et al.* 2011) and 230 000 records for eucalypts in Australia (Hui *et al.* 2014) from Australia’s Virtual Herbarium (avh.chah.org.au). To ensure maximal comparability between the acacia and eucalypt datasets, the data source and methods for cleaning and organizing the data followed protocols as set out in Richardson *et al.* (2011) and Robertson *et al.* (2016). We limited occurrence records used for further analyses to those representing native species with coordinates placing them in Australia, and excluded all hybrids and records only having genus names. We manually removed the invasive range records of eleven *Acacia* species with known invasive ranges within Australia, as they reflect human-mediated range in Australia rather than natural native range. The data editing and cleaning resulted in a list of c. 135 000 records for 1012 *Acacia* species and 145 000 records of 742 eucalypt species. The subset that matches the species in the phylogeny was used for all phylogenetic signal analyses.

Native range size and its aggregation structure have been identified as important predictors of the invasiveness of trees (e.g. Richardson *et al.* 2014), including for acacias and eucalypts (Gallagher *et al.* 2011; Hui *et al.* 2011, 2014). As such, we also included two metrics of native range size and aggregation. Specifically, for each species, the area-of-occupancy (AOO_*d*_) was calculated at seven different scales (*d* = 8, 16, 32, 64, 128, 256, 512 km) using a revised alpha-hull method (Hui *et al.* 2011) and the size of the convex hull (AOO_+_). The convex hull poses an upper bound to the alpha hulls, AOO_*d*_ = AOO_+_(1 − Exp[−*a*·*d*^2^^*b*^]), where a and b are the percolation intercept and exponent. Here, we choose two features of species’ geographical ranges in the analysis: the logarithm of an intermediate-scale range size log(AOO_128_) and percolation exponent *b*; (*b* = 0 indicate the species is filling up the extent of occupancy completely, while large *b* values indicates highly clustered distributions with many ‘holes’ in the range and potentially low rates of spread. For detailed explanations on the choice and meanings of these two metrics see Hui *et al.* (2011, 2014). We finally performed ANOVA tests for the two geographical ranges log(AOO_128_) and for the same set of species as on the phylogenetic signal tests.

## Results

**Figures 2.**
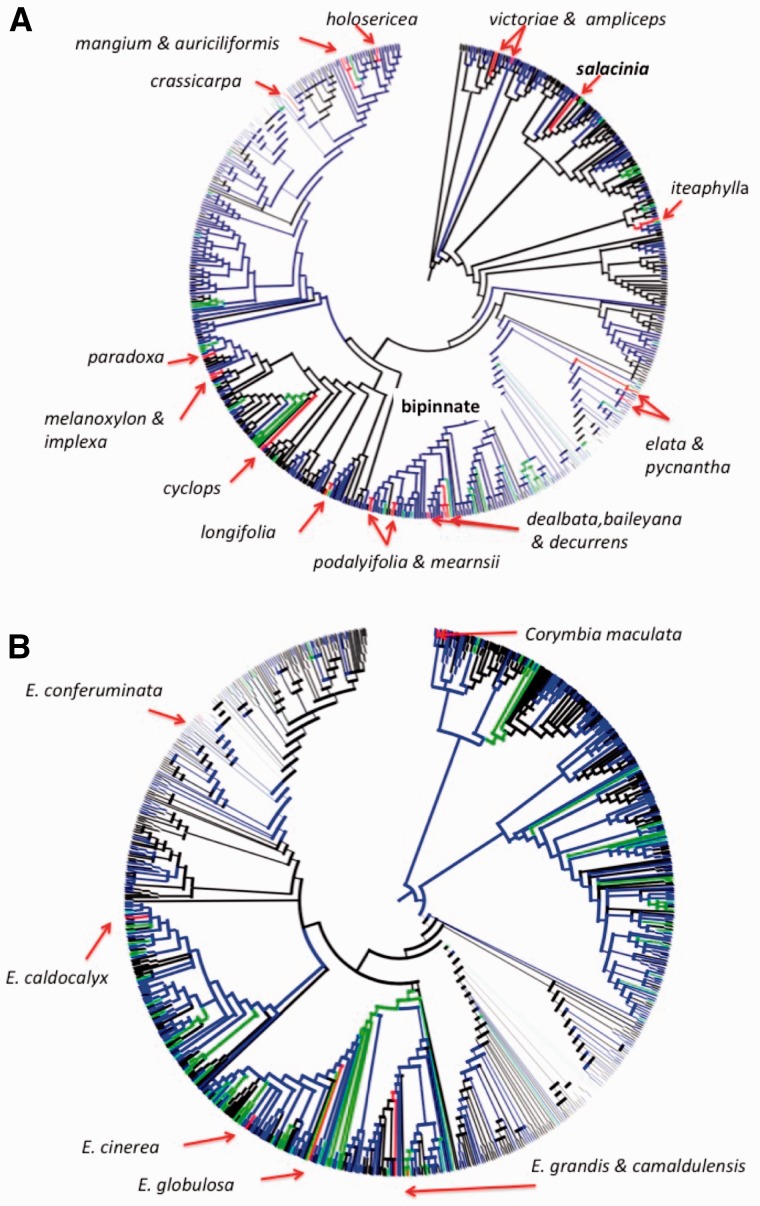
Phylogenetic trees of **(A)** *Acacia* and **(B)** eucalypts. Branches are coloured by introduction status. Red, invasive; Green, naturalized; Blue, introduced; and black, not introduced outside Australia. Interactive visualizations of these phylogenies can be found at *Acacia* (http://phylolink.ala.org.au/phylo/show/126#node/395373a92f9db36c18fc0845ebcf9db5) and *Eucalypts* (http://phylolink.ala.org.au/phylo/show/767#node/1a31ba3415717e8b 4ca f7e f 16d 73b72f).

**Figure 3:**
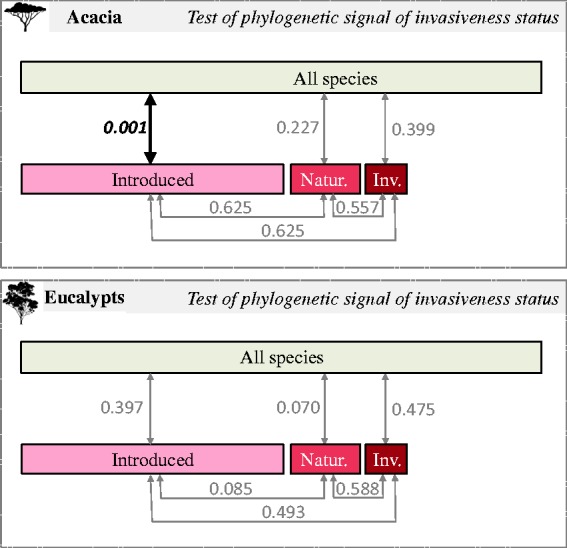
Analysis of phylogenetic signals of species introduction status as estimated by MPD index (with 999 randomizations). *P-*values: the species are significantly clustered (bold text) if *P*-value < 0.05 (and overdispersed if *P*-value > 0.95).

The status of each species along the INI continuum was mapped onto the phylogenies (Fig. 2). Visual inspection does not identify specific clades that are highly represented by introduction status (invasive, naturalized or introduced) in either the acacia or eucalypt phylogenies. These observations were supported by traditional phylogenetic signal analysis that found evidence of phylogenetic clustering of different species’ introduction statuses in only one instance (Fig. 3). For acacias, introduced species are more closely related than expected when analyzed as part of the entire dataset. However, there is no indication of clustering of any of the naturalized or invasive comparisons in *Acacia* when compared with a tree that does not contain non-introduced species. There is no significant signal of clustering in any of the eucalypt dataset comparisons (Fig. 3).

By using a novel measure of phylogenetic signal we found no evidence that invasive eucalypts or acacias were more ‘centralized’ in the phylogenetic network as they advance along the INI continuum (Table 1). Albeit statistically non-significant, there are clear trends in phylogenetic signal along the INI continuum: *Acacia* species become more marginalized in the phylogenic network (as indicated by an increase in Log(Mean), Log(Median) and Q.975; Table 1), whereas eucalypts become more centralized in the phylogenic network (as indicated by a decrease of Log(Mean), Log(Median) and Q.975; Table 1).

**Table 1.** Statistical analysis of range size and phylogenetic signal of *Acacia* and *Eucalypts*. Arrows highlight increasing and decreasing trends along the INI continuum.

*Acacia*	Introduced	Naturalized	Invasive	*F*_285,2_ ratio	*P*-value
Log (Range) ↑	10.937	11.459	12.223	6.487	**0.002**
Exponent ↓	0.820	0.627	0.580	5.047	**0.007**
Log (Mean) ↑	−3.511	−3.481	−3.437	1.128	0.325
Q.025	0.012	0.012	0.011	0.127	0.881
Q.975 ↑	0.057	0.058	0.061	1.301	0.274
Skewness	0.939	0.803	0.831	1.712	0.182
Kurtosis	5.870	5.422	5.561	0.554	0.575

*Eucalypts*	**Introduced**	**Naturalized**	**Invasive**	***F*_328,2_ Ratio**	***P*-value**

Log(Range) ↑	10.052	10.790	11.027	10.976	**0.000**
Exponent ↑	1.065	1.159	1.246	0.995	0.371
Log(Mean) ↓	−3.187	−3.245	−3.438	1.817	0.164
Q.025	0.006	0.004	0.005	0.813	0.444
Q.975 ↓	0.120	0.116	0.104	2.143	0.119
Skewness ↓	1.433	1.484	1.759	0.849	0.429
Kurtosis ↓	6.043	6.770	8.707	3.485	**0.032**

Neither the *Acacia* nor eucalypt datasets exhibits skewness in the pairwise genetic distances. The Kurtosis values for both acacias and eucalypts are >3, suggesting heavier tails (or more outliers) than predictions from a normal distribution. The significant increase of Kurtosis along the INI continuum for eucalypts indicates that the phylogenetic distance from invasive eucalypts to other non-invasive eucalypts is increasingly concentrated around the mean.

Regarding the native range size and spatial clustering analyses, we found that the introduction status of acacias is geographical-range related since both the log(AOO_128_) and the exponent of the occupancy show significant signals. Along the INI continuum, invasive acacias are detected to have larger ranges and shallower occupancy scaling (smaller exponents) than species that are only introduced or naturalized (Table 1). The results for the eucalypts similarly indicate that invasive species have larger range sizes, as log(AOO_128_) shows a significant signal, but the geographical exponent does not. These results generally indicate an increase in native range size along the INI continuum.

## Discussion

For both acacias and eucalypts we found no significant evidence for phylogenetic signal underlying the status of species along the INI continuum. In other words, naturalized and invasive taxa in these two groups appear to represent a random phylogenetic subset. However, introduced acacias do appear to represent phylogenetically more closely related species than would be expected by chance alone (also see Yessoufou *et al.* 2016). Using the full phylogeny we found no phylogenetic signal for the naturalized or invasive species groups.

In contrast, using only data from those acacias introduced outside Australia, Yessoufou *et al.* (2016) found phylogenetic signal underlying naturalized or invasive species groups. One reason that can explain the differences with our result is the fact that these authors transformed the *Acacia* phylogeny into a chronogram without fossil dating information, whereas we used the observed genetic distances between species. Indeed, a chronogram estimated without fossil information assumes homogenous mutational rates across all branches, which can bias estimates of the evolutionary distance separating taxa. This may account for the discrepancies between our findings based on raw branch lengths and those of Yessoufou *et al.*’s (2016) and highlights the sensibility of phylogenetic signal estimates to the type of phylogeny used.

The lack of phylogenetic signal in *Acacia* and eucalypts for all cases except for introduced species could be attributable to four main factors. First, different traits may drive invasiveness in different parts of the phylogeny (e.g. some clades could be more invasive because of high seed production, whereas taxa in other clades could be invasive because of good competitive abilities). Second, traits driving invasiveness may follow specific modes of evolution that cannot be captured by simple phylogenetic signals (e.g. Ornstein-Uhlenbeck model of evolution with multiple optima). Third, human mediated propagule pressure may be more important than the classical invasiveness traits, as human selection for alien species is often related to species economical (such as wood quality or tannin content) or ornamental values (such as large flowers or large canopies) which may produce phylogenetic signals that are not related to species invasiveness (potentially explaining why we found phylogenetic signal for introduced *Acacia* species). Lastly, because not all species were introduced at the same time, and not all introduced species become invasive with the same rate, it is likely that some of the taxa that are now labeled ‘introduced’ may become invasive in the future. This ‘invasion debt’ (Rouget *et al.* 2016) may thus bias our introduction status estimate.

Despite the lack of significant phylogenetic signal in the INI continuum in *Acacia* and eucalypts, there are trends in the biogeographic attributes of these two groups. Range size increases in both *Acacia* and eucalypts from introduced to naturalized to invasive (Table 1). Although the native range size for both *Acacia* and eucalypt species increases with introduction status, the rate of expansion as inferred from the percolation exponent is different, decreasing for *Acacia* (higher rate of spread) but increasing for eucalypts (lower rate of spread) along the INI continuum (Table 1; also see Hui *et al.* 2011, 2014). Some phylogenetic metrics did show a non-significant trend along the INI continuum, with *Acacia* species become more marginalized in the phylogenic network (increasing Log(Mean), Log(Median) and Q.975 in Table 1), whereas eucalypts become more centralized in the phylogenic network (reducing Log(Mean), Log(Median) and Q.975 in Table 1). This means that, in both lineages, centralization in the phylogenetic network (i.e. a network of the phylogenetic distance matrix) is associated with reduced rate of spread.

We detected opposite phylogenetic trends for the two clades along the INI continuum. First, classic phylogenetic methods, such as the MPD methods, are inconclusive in detecting signals and trends (but see Yessoufou *et al.* 2016). These phylogenetic trends have a weaker predictive power of species invasiveness than native range/niche size (and adult plant height although not tested here; Hui *et al.* 2011, 2014). Second, even though it may be impossible to identify invasive lineages, a ‘network’ view of phylogenetic trees (a network oriented interpretation of phylogenetic distances) provides an alternative way of viewing invasiveness. We found that invasive species as located at the margin of the network, correlated with their faster spreading ability in their native ranges. Non-invasive species are located at the center of the phylogenetic network. This correlates with the slower spreading species in the native ranges.

## Conclusions

It is unclear whether phylogenetic history plays a major role in the invasiveness of acacias and eucalypts. However the trends along the INI continuum towards invasiveness suggest (i) smoother geographic distributions in more invasive acacias (decreasing exponents) but more clustered geographic distributions in more invasive eucalypts (increasing exponents), (ii) location of invasive species on the edges of phylogenetic networks (increasing log mean, median and Q.975, but decreasing marginality with eucalypt invasiveness) and (iii) phylogenetic distance between invasive and non-invasive species are trending toward the mean pairwise distance (increased skewness and kurtosis) in eucalypt but not in *Acacia.* This paints the picture of an *Acacia* clade containing invasive species that have smoother and increasing distributions, and that are located closer to the edges of phylogenetic networks than the eucalypt invasive species. Indeed the eucalypts may be less invasive in general because these traits lessen along the INI continuum such that fewer eucalypts than *Acacia* species have the capacity to progress beyond the naturalization stage in the INI continuum.

By examining the signals of phylogeny and range structures in overcoming the barriers along the INI, we are testing whether these factors are largely the same or distinct from each other. It is unknown whether the biological and ecological factors behind phylogeny, range structures and invasiveness are similar. Nonetheless, we conclude that factors driving range (dynamics) and invasiveness could largely be the same, and that factors driving phylogeny and invasiveness are only marginally shared (resulting in weak signal).

This information may be helpful for screening species for invasiveness (risk assessment) before introducing them outside of Australia. *Acacia* and eucalypts are not the only woody tree groups that are invasive outside their native Australian ranges—e.g. members of the Proteaceae also problematic weeds in many parts of the world (Moodley *et al.* 2013). The differential patterns seen in *Acacia* and eucalypts, and other Australian lineages, can be used in a comparative framework to test the notion that evolution in Australia has acted as a factory to produce a collection of trees that are highly invasive when moved by humans to many other parts of the world.

## Sources of Funding

This article had its origin at a workshop on ‘Evolutionary dynamics of tree invasions’ hosted by the DST-NRF Centre of Excellence for Invasion Biology (C•I•B) in Stellenbosch, South Africa, in November 2015. Funding for the workshop was provided by the C•I•B, Stellenbosch University (through the office of the Vice Rector: Research, Innovation and Postgraduate Studies), and the South African National Research Foundation (DVGR grant no. 98182). This article includes work done by J.M. while serving at the National Science Foundation. The views expressed in this article do not necessarily reflect those of the National Science Foundation or the United States Government.

## Contributions by the Authors

J.M., D.R. and C.H conceived the basis of the article. J.M., C.H., J.L.R., A.T. and LA.G. performed data analyses. JM led the writing. All authors contributed to the writing of the article.

## Conflicts of Interest Statement

None declared.
